# Chaihu Shugan San ameliorated cognitive deficits through regulating gut microbiota in senescence-accelerated mouse prone 8

**DOI:** 10.3389/fphar.2023.1181226

**Published:** 2023-05-15

**Authors:** Zhiyue Li, Qiang Zeng, Shengquan Hu, Zhanyan Liu, Shuting Wang, Yu Jin, Limin Li, Hanlin Ou, Zhengzhi Wu

**Affiliations:** ^1^ The First Affiliated Hospital of Shenzhen University, Shenzhen Second People’s Hospital, Shenzhen, China; ^2^ Academician Workstation, Ningbo College of Health Sciences, Ningbo, China; ^3^ The Eighth Affiliated Hospital of Sun Yat-Sen University, Shenzhen, China; ^4^ State Key Laboratory of Bio-Fibers and Eco-Textiles, Collaborative Innovation Center of Marine Biobased Fiber and Ecological Textile Technology, College of Materials Science and Engineering, Qingdao University, Qingdao, China

**Keywords:** mild cognitive impairment, gut microbiota, neural injury, Chaihu Shugan San, senescence-accelerated mouse prone 8

## Abstract

**Background:** Traditional Chinese medicines exhibit promising preventive effects on Alzheimer’s disease. Chaihu Shugan San (CSS) is a well-known traditional herbal formula whose several kinds of ingredients have the potential of ameliorating Alzheimer’s disease. The present study aimed to evaluate the effects of CSS on the microbiota–gut–brain axis and cognitive deficits of senescence-accelerated mouse prone 8 (SAMP8) mice as well as investigate the underlying mechanisms.

**Methods:** Thirty 5-month-old SAMP8 mice were randomly divided into the model group (SAMP8), CSS low-dose treatment group (CSSL), and CSS high-dose treatment group (CSSH). Ten SAMR1 mice were used as the normal control, and ten SAMP8 mice treated with donepezil were used as the positive control of cognitive function. CSS was orally administrated to SAMP8 mice for 8 weeks. The Morris water maze test was used to evaluate cognitive function. Histological staining was used to observe neuronal injury and Aβ deposition. Transmission electron microscopy was used to observe the synaptic ultrastructure. 16S rRNA gene analysis was performed to measure the changes in intestinal microbiota.

**Results:** The results showed that CSS significantly improved the learning function and memory deficits of aged SAMP8 mice in the Morris water maze examination. CSS ameliorated neuronal injury, synaptic injuries, and Aβ deposition in the brain of SAMP8 mice. In addition, CSS also significantly improved microbiota composition in terms of elevating *Lactobacillus reuteri* and decreasing *Staphylococcus xylosus* in the feces of aged SAMP8 mice.

**Conclusion:** These findings suggested that CSS might have a preventive potential for cognitive deficits in aging through regulating gut microbiota, which paved the way for the application of CSS for prevention and therapeutic purposes for mild cognitive impairment as well as Alzheimer’s disease.

## 1 Introduction

Alzheimer’s disease (AD) is a progressive neurodegenerative disease associated with structural changes in the brain that reflect advanced aging (Wang et al., 2022). Mild cognitive impairment (MCI) is considered an intermediate stage between normal aging and dementia. Amnestic MCI (aMCI), the most common subtype of MCI, involves memory impairment that is more likely to progress to typical AD (Cong et al., 2023). The typical clinical symptom of AD is cognitive dysfunction, which causes poor life quality and a heavy burden to patients. The incidence of AD has been rising and is estimated to rise globally up to 152.8 million cases by 2050 as a result of the increasing number of elderly people (GBD, 2019 Dementia Forecasting Collaborators, 2022). The pathogenesis of AD has not been clearly elaborated, but is recognized generally to be associated with specific neurotransmitters deficiency, neuroinflammation, synaptic loss, neuronal death, and abnormal proteinaceous accumulation in neurons. The major pathological changes in the AD brain consisted of the deposition of insoluble amyloid β (Aβ) to form senile plaques and the aggregation of phosphorylated tau protein (p-tau) to form neurofibrillary tangles (NFTs) (Guo et al., 2020).

A diverse combination of bacteria settle in the gut, and the balance of intestinal flora plays an important role in maintaining body health. Previous studies suggested that intestinal microbiota was associated with neuropsychiatric disorders, such as Parkinson’s disease and depression (Rieder et al., 2017). Recently, plenty of studies have shown that intestinal microbiota contributed to the pathogenesis of AD by regulating the host immune system, amino acid metabolism, neurotransmitters secretion, and endocrine system. The composition and diversity of intestinal microbiota were very different between AD patients and normal people (Vogt et al., 2017; Zhuang et al., 2018). It was found that *Bacteroides* and *Lachnospiraceae* decreased while *Actinobacteria* and *Ruminococcus* increased in the feces of AD patients (Zhuang et al., 2018). Notably, emerging pieces of evidence supported that the dysbiosis of intestinal microbiota during the aging process impaired the intestinal barrier, increased intestinal permeability and released pro-inflammatory products, led to inflammation, and eventually induced microglia activation to participate in AD progression (Cattaneo et al., 2017). Supplements of probiotics containing *Lactobacillus acidophilus*, *Bifidobacterium lactis*, and *Bifidobacterium longum* could improve the memory and learning function of transgenic mice as well as the rats that received an intrahippocampal injection of Aβ_42_ (Athari Nik Azm et al., 2018; Abraham et al., 2019). Therefore, maintaining the balance of intestinal microbiota may partly attenuate the progress of AD and is probably an available strategy to improve cognitive deficits of AD.

Chaihu Shugan San (CSS) is a well-known traditional herbal formula that has the function of soothing liver-qi and relieving depression according to the theory of traditional Chinese medicine (TCM). CSS has been used for hundreds of years and is traditionally used for the treatment of depression and menopause-related symptoms (Wang et al., 2012). Modern pharmacological studies demonstrated that CSS not only ameliorates gastrointestinal diseases and inhibits hepatic injury but also ameliorates memory dysfunction induced by D-galactose and Aβ_1-42_ (Zhao et al., 2012; Qin et al., 2013; Jia et al., 2017). A previous study showed that CSS had a protective effect against Aβ-induced neural cell death through the Akt signaling pathway (Zeng et al., 2019). It was discovered that several kinds of ingredients in CSS had potential therapeutic effects on AD. Saikosaponin C, one of the bioactive components in *Radix Bupleuri*, could significantly inhibit Aβ release and abnormal tau phosphorylation, promote neurite outgrowth, as well as increase synaptic marker protein such as synaptophysin expression (Lee et al., 2016). Paeoniflorin, a major bioactive constituent in *Radix Paeoniae*, could significantly alleviate amyloidogenesis, inhibit astrocyte activation, and decrease inflammatory response in a transgenic mouse model of AD (Zhang et al., 2015; Kong et al., 2020). Moreover, the antidepressant effect of CSS was involved in improving gut microbiota dysbiosis and related metabolic disturbance (Yu et al., 2020). However, the role of the gut microbiota in the pathogenesis of AD and the effect of CSS on cognitive function remain unknown. Therefore, the present study aimed at investigating the ameliorative effect of CSS on cognitive deficits and its underlying mechanisms through regulating intestinal microbiota and inhibiting inflammation in senescence-accelerated mouse prone 8 (SAMP8). This study provided more information on the interplay between gut microbiota and the anti-dementia effect of CSS.

## 2 Materials and methods

### 2.1 Preparation of CSS aqueous extract

The raw herbs were purchased from Tong Ren Tang Chinese Medicine (Beijing, China) and were authenticated in accordance with the requirement of the Chinese Pharmacopeia 2015. The aqueous extracts were prepared as in the previous study. In brief, the herbs were cut into pieces and extracted with boiling distilled water (1:10, w/v) under reflux twice, 2 h each time. Then the CSS water extract (CSSW) was filtered under reduced pressure and concentrated by rotary evaporation. Finally, the extract was lyophilized for use. The extraction yield of CSSW was 32.5% (w/w). The chemical analysis of CSSW was the same as in the previously published study by Zeng et al. (2019).

### 2.2 Animals and treatment protocols

The senescence-accelerated mouse prone 8 (SAMP8, male, 5 months old) and senescence-resistant mouse (SAMR1) were obtained from the Department of Laboratory Animal Science of Peking University Health Science Center [SCXK (Jing) 2016-0010]. All animal experiment designs and protocols were approved by the Animal Experimentation Ethics Committee of the Shenzhen Second People’s Hospital. Thirty SAMP8 mice were randomly divided into three groups (*n* = 10 for each group): the model group (SAMP8), CSS low-dose treatment group (CSSL), and CSS high-dose treatment group (CSSH). The mice in these three groups were orally administered distilled water, 2.1 g/kg/day CSSW, and 4.2 g/kg/day CSSW, respectively. On the other hand, 10 SAMR1 mice were used as normal control, and they were administered with distilled water instead. A total of 10 SAMP8 mice administered with donepezil (1 mg/kg/day) were used as the positive control of cognitive function. All drugs were given once a day for 8 weeks. All animals were kept under standard laboratory conditions with controlled light (12 h light/12 h dark) and an ambient temperature of 22°C ± 2°C. They were housed in three or four per cage with free access to water and food.

### 2.3 Morris water maze test

The Morris water maze test (MWMT) was used to evaluate cognitive functions, such as learning and spatial memory ability, and was performed as previously described. In brief, the MWMT was conducted in a circular swimming pool (120 cm diameter and 50 cm height) containing a hidden platform (10 cm diameter), which was placed in the lower left quadrant and submerged 1 cm below the water surface. The behavioral test consisted of a positioning navigation experiment and a spatial probe experiment. In the positioning navigation experiment, mice were trained to find the hidden platform. Each mouse was placed in the water from the four quadrants of the pool respectively and given 60 s to find the hidden platform. If the mice could not locate the platform within 60 s, they were gently guided to the platform and allowed to remain on it for 15 s. The mice were subjected to three trials per day for five consecutive days. The time taken to find the platform (escape latency) was recorded as the indicator for reflecting learning skills. A spatial probe trial was performed to the evaluate memory skill of the mice on the sixth day of the test. The platform was removed, and each mouse was placed in the water from the upper right quadrant and allowed to swim freely to the removed platform within 120 s. The latency to the first entry of the hidden platform and the times of crossing the platform were measured as indicators for reflecting spatial memory skills.

### 2.4 Nissl staining

Nissl staining was used to detect neuron morphology, as reported previously. After the neurobehavioral test, the brain tissues were collected. The left hemisphere was frozen in liquid nitrogen and used for transmission electron microscopy imaging. The right hemisphere was fixed in 4% polyformaldehyde, and routine dehydration, embedding, and microtome section were performed. After the sections were dewaxed and rehydrated, they were stained with cresyl violet (Beyotime Biotechnology, Shanghai, China) for 5 min. The Nissl body displayed royal purple color. The photos of the entorhinal cortex and hippocampus were captured.

### 2.5 Immunohistochemistry staining of β-amyloid

After the sections were dewaxed and rehydrated, antigen heat retrieval was performed in citrate buffer. After washing with PBS, 0.5% TritonX-100 was used to expose the cell antigen at room temperature. The sections were rinsed with PBS, and 0.3% hydrogen peroxide was used to inactivate peroxidase in the tissue. Then 10% normal goat serum (Hyclone, United States) was added to the block for 1 h, and the primary antibody against mouse β-amyloid (1:50, Zen Bioscience, China) was incubated with the sections overnight at 4°C. After washing three times with PBS, goat secondary antibody against rabbit immunoglobulins coupled with peroxidase (1:50, Abcam, United States) were added to slides and incubated for 1 h at room temperature. The chromogenic reaction was performed by Dako REAL™ DAB chromogen diluted in substrate buffer (Dako, Denmark). Finally, the nuclei were counterstained in hematoxylin. The photos of brown positive β-amyloid were captured.

### 2.6 Transmission electron microscopy

The synaptic ultrastructure of the hippocampal CA1 region was observed by transmission electron microscopy. The left brain tissue was fixed in 4% glutaraldehyde for 24 h and cut into 1-mm dice. The samples were fixed in 1% osmium tetroxide for 1 h, washed in phosphate-buffered saline (PBS) twice, dehydrated in a graded series of acetone, and then embedded in Epon-812 reagent. The ultrathin sections were cut into 50 nm thick slices and desiccated. Then the sections were stained with uranyl acetate (Ted Pella Inc., CA, United States) and lead citrate (Beijing Zhongjingkeyi Technology Company, Beijing, China) for 10 min at room temperature. After rinsing and drying, the synaptic ultrastructure of the hippocampal CA1 region was observed under JEM-1400 plus transmission electron microscope (JEOL, Japan).

### 2.7 Intestinal microbiota analysis

Fresh feces samples were collected after the behavioral test and used for 16S rRNA gene analysis of microbiota profiling with barcoded amplicons from the V3–V4 region. The DNA extraction, PCR amplification, and sequencing were conducted by Majorbio Bio-Pharm Technology Co., Ltd. (Shanghai, China). In brief, genomic DNA was extracted from feces samples using the E.Z.N.A.^®^ soil DNA Kit (Omega Bio-tek, Norcross, GA, United States) according to the manufacturer’s instructions. DNA concentration and purity were determined with NanoDrop 2000 UV-vis spectrophotometer (Thermo Scientific, Wilmington, United States). The hypervariable region V3–V4 of the bacterial 16S rRNA gene was amplified with primers 338F (5′-ACT​CCT​ACG​GGA​GGC​AGC​AG-3′) and 806R (5′-GGACTACHVGGGTWTCTAAT-3′) by an ABI GeneAmp^®^ 9700 PCR thermocycler (ABI, CA, United States). The PCR amplification of the 16S rRNA gene was carried out in triplicates as follows: initial denaturation at 95°C for 3 min, 27 cycles of denaturing at 95°C for 30 s, annealing at 55°C for 30 s, and extension at 72°Cfor 45 s, followed by single extension at 72°C for 10 min and end at 4°C. The PCR reaction system contained 5× FastPfu buffer 4 μL, 2.5 mM dNTPs 2 μL, forward primer (5 μM) 0.8 μL, reverse primer (5 μM) 0.8 μL, FastPfu DNA Polymerase 0.4 μL, DNA template 10 ng, and, finally, ddH_2_O up to 20 μL. The PCR product was extracted from 2% agarose gel and purified using the AxyPrep DNA Gel Extraction Kit (Axygen Biosciences, Union City, CA, United States) according to the manufacturer’s instructions and quantified using Quantus™ Fluorometer (Promega, United States).

Purified amplicons were pooled in equimolar and paired-end sequenced on an Illumina MiSeq PE300 platform (Illumina, San Diego, United States). The sequencing data were analyzed using the free online platform of Majorbio Cloud Platform (www.majorbio.com). The raw 16S rRNA gene sequencing reads were demultiplexed, quality-filtered, and merged according to the following criteria: (i) the 300 bp reads were truncated at any site receiving an average quality score of <20 over a 50 bp sliding window, and the truncated reads shorter than 50 bp and reads containing ambiguous characters were discarded; (ii) only overlapping sequences longer than 10 bp were assembled according to their overlapped sequence; (iii) Samples were distinguished according to the barcode and primers, and the sequence direction was adjusted. Operational taxonomic units (OTUs) with 97% similarity cutoff were clustered using UPARSE version 7.1 (Edgar, 2013), and chimeric sequences were identified and removed. The taxonomy of each OTU representative sequence was analyzed by the RDP Classifier algorithm against the Silva 16S rRNA database using a confidence threshold of 0.7.

### 2.8 Statistics analysis

The data in the present study were expressed as means ± SEM and analyzed with GraphPad Prism 5.0 (GraphPad Software, CA, United States). The differences among the groups were statistically analyzed by one-way ANOVA with the Bonferroni test. The Spearman correlation analysis between the relative abundance of *Lactobacillus reuteri* and the behavior test was performed. In all comparisons, *p* < 0.05 was considered statistically significant.

## 3 Results

### 3.1 CSS improved learning and memory function of SAMP8 mice

**FIGURE 1:**
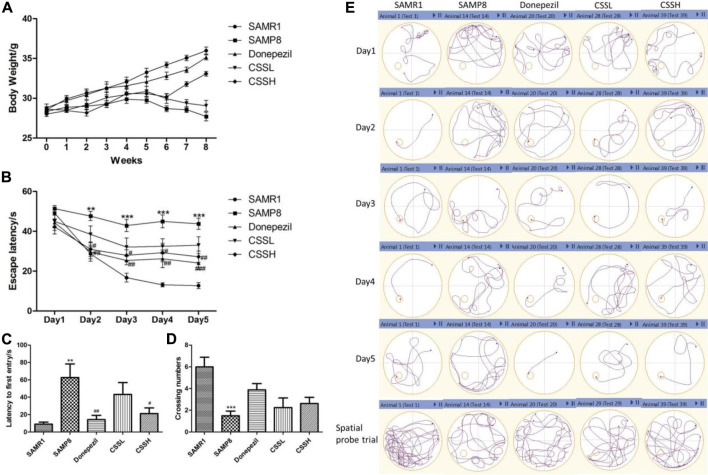
CSS improved the weight loss, learning, and memory function of SAMP8 mice. **(A)** Effect of CSS on weight loss in SAMP8 mice. **(B)** Effect of CSS on escape latency in the positioning navigation experiment. **(C)** Effect of CSS on latency to first entry in the spatial probe trial. **(D)** Effect of CSS on crossing number in the spatial probe trial. **(E)** Representative examples of behavior trajectories in positioning navigation experiment and spatial probe experiment. Data were expressed as mean ± SEM (*n* = 6–8). ***p < 0.001, **p < 0.01 showed significant difference compared with the SAMR1 group at each time point. ^#^ *p* < 0.05, ^##^ *p* < 0.01, and ^###^ *p* < 0.001 showed a significant difference compared with the SAMP8 group at each time point.

As shown in Figure 1A, SAMR1 mice had a stable increase in body weight in the experiment period. The body weight of SAMP8 mice did not increase. They even had weight loss in the fifth week. On the contrary, the SAMP8 mice orally administered with CSS showed ameliorated body weight loss than SAMP8 mice administered with distilled water.

The MWMT was used to evaluate cognitive functions such as learning and spatial memory ability (Figures 1B–E). The mice in all the groups took less time to find the platform (escape latency) via 5-day training. The escape latency of SAMP8 model mice was significantly longer than that of SAMR1 mice on days 2, 3, 4, and 5. However, CSS treatment could significantly shorten escape latency in SAMP8 mice, indicating that CSS could improve learning ability (Figure 1B). On the other hand, the SAMP8 mice showed a significant increase in latency to the first entry and a decrease in the number of crossing platforms compared with SAMR1 mice. CSS treatment in high doses could significantly shorten the latency to the first entry and showed a tendency to increase the platform crossing number in SAMP8 mice, demonstrating that CSS could improve spatial memory function in SAMP8 mice (Figures 1C, D). The representative examples of behavior trajectories in water maze test were shown in (Figure 1E).

### 3.2 CSS ameliorated neuronal injury and Aβ deposition in the brain of SAMP8 mice

**FIGURE 2:**
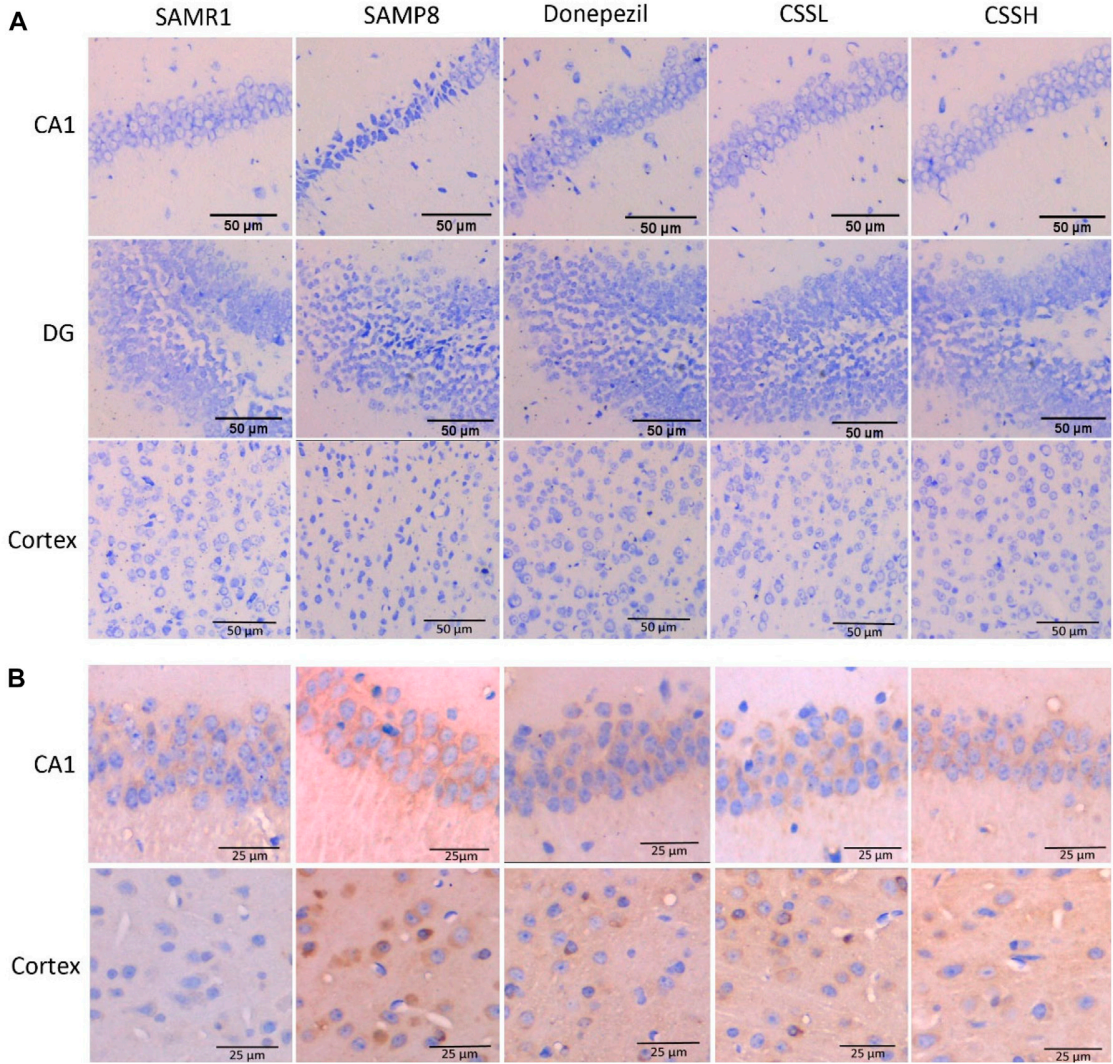
CSS ameliorated neuronal injury and Aβ deposition in the brain of SAMP8 mice. **(A)** Effect of CSS on neuronal injury by Nissl staining. The neurons were loose with cytoplasmic shrinkage in the CA1, DG of the hippocampus, and the brain cortex subregion of SAMP8 model mice, while CSS administration inhibited abnormal cell morphology changes. Scale bar: 50 μm. **(B)** Effect of CSS on Aβ deposition by immunohistochemistry staining. The Aβ deposition was observed in the hippocampal CA1 subregion and cortex of SAMP8 mice, while CSS prevention reduced Aβ deposition significantly. Scale bar: 25 μm.

The reduced Nissl body was observed in damaged neurons. As shown in Figure 2A, the neurons showed normal neuronal cell morphology with clear edges in the CA1 region of the hippocampus and cortex of SAMR1 mice. Compared to those in SAMR1 mice, the neurons in the CA1, dentate gyrus (DG) of hippocampus and brain cortex region of SAMP8 model mice were loose with shrunk cytoplasm, and the number of Nissl staining positive cells was significantly decreased, indicating an increase in injured neurons. However, CSS treatment could increase Nissl staining positive cells, indicating that CSS could inhibit neuronal injury in the brain of SAMP8 mice.

Moreover, Aβ deposition was observed in the hippocampal CA1 region and cortex of SAMP8 mice (Figure 2B). However, Aβ deposition was significantly reduced after CSS and donepezil intervention. The inhibitory effects of CSS on Aβ deposition were similar to the effects of donepezil.

### 3.3 CSS inhibited synaptic structure damage in the hippocampus of SAMP8 mice

**FIGURE 3:**
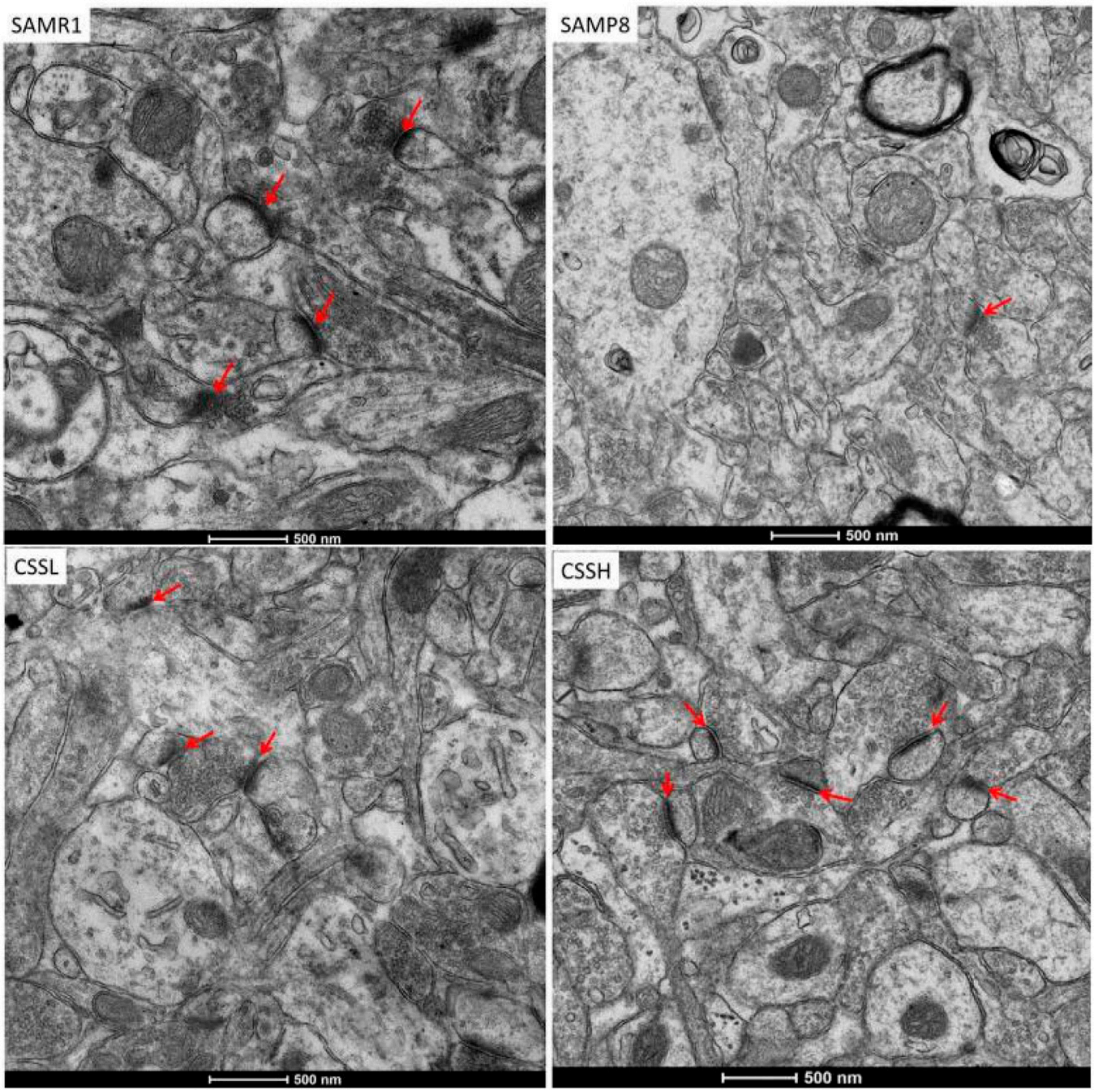
Representative transmission electron micrographs of synaptic structure in the hippocampal CA1 area. Red arrow showed synaptic structure. CSS treatment could ameliorate the damaged synapses. Scale bar: 500 nm.

As shown in Figure 3, the hippocampal cortex in SAMR1 mice showed well-preserved synaptic ultrastructure, clear presynaptic and postsynaptic parts of synapses with normal structure, as well as a large number of synaptic vesicles in axonal-end bulbs. Remarkable damage was observed in the hippocampal cortex in SAMP8 mice. Specifically, the synapse showed substantially enlarged presynaptic parts, reduced content of synaptic vesicles, and their abnormal clumping in the vicinity of the synaptic cleft. However, the damaged synapses were ameliorated after CSS treatment. The presynaptic and postsynaptic parts of the synapses and the content of synaptic vesicles were restored after CSS treatment. The results demonstrated that CSS could inhibit synaptic structure damage in the hippocampus of SAMP8 mice.

### 3.4 CSS regulated intestinal microbiota in SAMP8 mice

**FIGURE 4:**
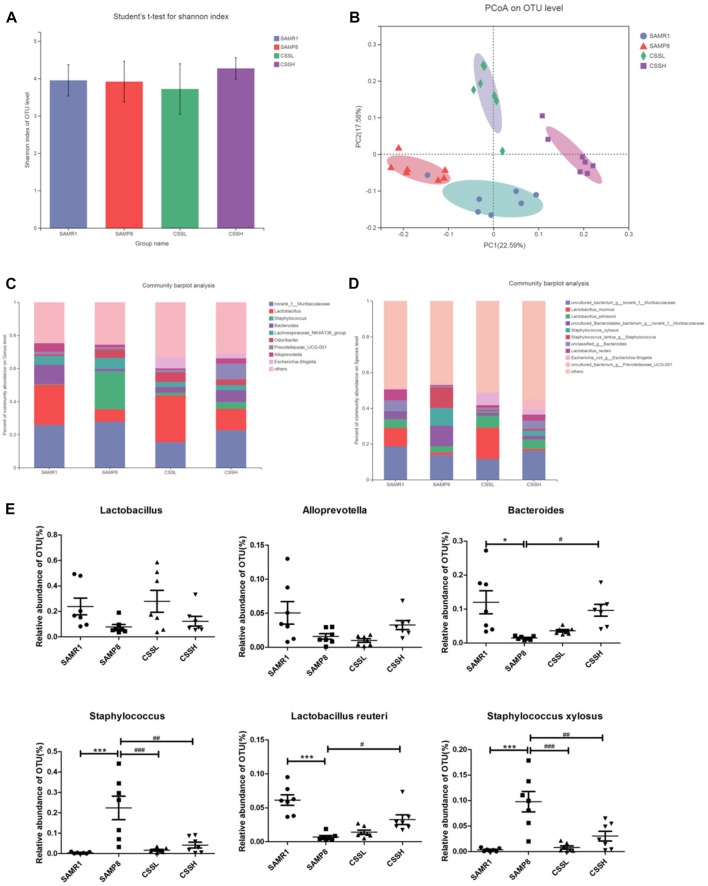
CSS regulated intestinal microbiota in SAMP8 mice. **(A)** Student’s *t*-test for Shannon index of alpha diversity. **(B)** PCoA plots at the operational taxonomic unit (OTU) level. **(C)** Community barplot analysis of relative abundance at the genus level. **(D)** Community barplot analysis of relative abundance at the species level. **(E)** Differences in the relative abundance of OTU level among the four groups. The data were analyzed by one-way ANOVA with the Bonferroni test. ***p < 0.001 and *p < 0.05 showed a significant difference compared with the SAMR1 group. ^#^ *p* < 0.05, ^##^ *p* < 0.01, and ^###^ *p* < 0.001 showed a significant difference compared with the SAMP8 group.

To assess the role of gut microbiota alteration in AD pathogenesis, the sequence of 16S rRNA gene V3–V4 region in fecal samples was analyzed. The microbial alpha diversity reflects species richness and evenness. As shown in Figure 4A, the Shannon index was measured and was not significantly different among the groups, indicating that CSS administration did not improve the total species diversity of SAMP8 mice. As shown in Figure 4B, the cluster from the SAMP8 group samples was different from that of the SAMR1 group samples by principal coordinate analysis (PCoA). Similarly, the cluster from the CSS treatment group samples was also different from that of the SAMP8 group samples. The results indicated that there were differences in the microbiota composition among the four groups. Moreover, community barplot analysis was conducted to illustrate the difference in the microbiota composition. As shown in Figures 4C, E, at the genus level, there was a decrease in *Lactobacillus* (SAMP8 vs. SAMR1 = 7.4% vs. 24.3%), *Bacteroides* (SAMP8 vs. SAMR1 = 1.5% vs. 11.7%),and *Alloprevotella* (SAMP8 vs. SAMR1 = 1.6% vs. 5.2%) and an increase in *Staphylococcus* (SAMP8 vs. SAMR1 = 22.9% vs. 0.4%) in the SAMP8 group compared with the SAMR1 group. However, CSS treatment reversed the decrease in *Lactobacillus* (CSSH vs. SAMP8 = 13.0% vs. 7.4%), *Bacteroides* (CSSH vs. SAMP8 = 7.2% vs. 1.5%), and *Alloprevotella* (CSSH vs. SAMP8 = 3.2% vs. 1.6%) and reversed the increase in *Staphylococcus* (CSSH vs. SAMP8 = 4.0% vs. 22.9%) in CSSH group compared with the SAMP8 group. As shown in Figures 4D, E, at the species level, the results showed that the relative abundance of *L. reuteri* decreased significantly (SAMP8 vs. SAMR1 = 0.7% vs. 6.2%) and the relative abundance of *Staphylococcus xylosus* increased significantly in the SAMP8 group (SAMP8 vs. SAMR1 = 10.0% vs. 0.3%). However, CSS treatment raised the relative abundance of *L. reuteri* significantly (CSSH vs. SAMP8 = 3.4% vs. 0.7%) and reduced the relative abundance of *S. xylosus* (CSSH vs. SAMP8 = 2.9% vs. 10.0%) compared to the SAMP8 group. All these data demonstrated that CSS could modulate the intestinal microbiota of SAMP8 mice.

### 3.5 *Lactobacillus reuteri* abundance change was in correlation with cognitive performance in SAMP8 mice

**FIGURE 5:**
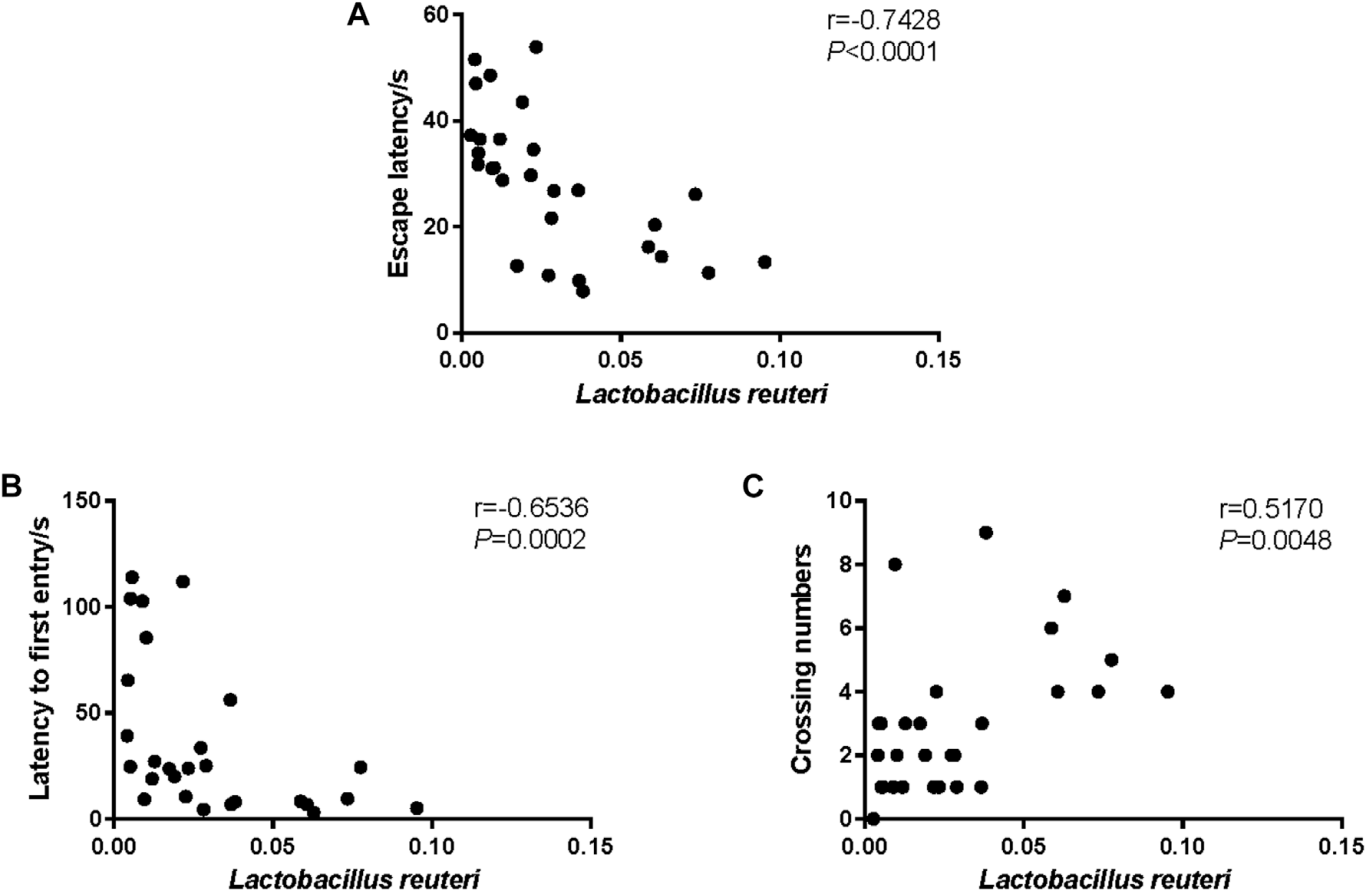
Correlation between *Lactobacillus reuteri* and the behavior performance. **(A)** The Spearman correlation analysis between the relative abundance of *L. reuteri* and the escape latency. **(B)** The Spearman correlation analysis between the relative abundance of *L. reuteri* and latency to first entry. **(C)** The Spearman correlation analysis between the relative abundance of *L. reuteri* and crossing numbers. The correlation coefficients (*r*) and the *p*-value are shown in the top right.

In order to identify a marker of cognitive performance in SAMP8 mice, the Spearman correlation analysis was conducted between *L. reuteri* and the behavior performance. As revealed in Figure 5, we observed that the relative abundance of *L. reuteri* was in significantly negative correlation with the escape latency (Figure 5A, *r* = −0.7428, *p* < 0.0001) and the latency to the first entry (Figure 5B, *r* = −0.6536, *p* = 0.0002), and it was in significantly positive correlation with the crossing numbers (Figure 5C, *r* = 0.5170, *p* = 0.0048).

## 4 Discussion

A large number of researches have demonstrated that the microbiota–gut–brain axis played an essential role in the progression of aging-related cognitive deficits. However, it is still a big challenge to find effective agents for the prevention and treatment of cognitive impairments in aged people. Recent studies from different research teams have exposited several probiotics, including *B. lactis*, *Lactobacillus casei*, *B. bifidum*, and *L. acidophilus*, had neuroprotective effects on aging-related cognitive dysfunctions in clinical and preclinical studies (Akbari et al., 2016; Athari Nik Azm et al., 2018; Tamtaji et al., 2019; Yang et al., 2020). In the present study, we observed similar results that the imbalance of the microbiota in terms of decreasing probiotics of the *Lactobacillus* family, especially *L. reuteri*, was correlated with cognitive decline in aged SAMP8 mice. Furthermore, oral administration of CSS, a traditional Chinese medicine, for 8 weeks significantly improved the memory deficits and the learning function of aged SAMP8 mice in the Morris water maze examination. CSS ameliorated neuronal injury, synaptic injury, and Aβ deposition in the brain of SAMP8 mice. In addition, CSS also significantly improved microbiota composition in terms of elevating *L. reuteri*, and decreasing *S. xylosus* in the feces of aged SAMP8 mice. These findings, to our best knowledge, suggested for the first time that CSS may have a preventive potential for cognitive deficits in SAMP8 mice by targeting the gut microbiota. The current study paved the way for the application of CSS against aging-associated cognitive impairments, including Alzheimer’s disease.

CSS is a classical traditional herbal formula used for hundreds of years since the Ming Dynasty. CSS is famous for its function of soothing liver-qi and relieving depression. According to the theory of TCM, the herbs contained in CSS, such as *Bupleurum chinense* DC. (Chaihu), *Paeonia lactiflora* Pall. (Baishao), *Ligusticum chuanxiong* Hort. (Chuanxiong), and *Cyperus rotundus* L. (Xiangfu), are attributed to the liver meridian, indicating that the CSS functions by targeting the liver. Recent studies revealed that the contribution of the liver to Aβ clearance and regulation of brain energy metabolic disturbances has attracted interest (Huang et al., 2022). The decline of hepatic Aβ clearance during aging could be involved in AD development, which provides new opportunities for AD treatment (Cheng et al., 2023). These findings implied the possibility of CSS, a well-known formula with the function of soothing liver-qi, ameliorating cognitive deficits through liver modulation.

SAMP8 mice are characterized by an early onset of age-related disorders accompanied by neuronal injury as early as 6 months old (Pacesova et al., 2022). Among all SAMP mouse strains, SAMP8 mice show senescence deterioration in cognitive function compared with senescence-resistant (SAMR1) control mice (del Valle et al., 2012). SAMP8 is a widely used mouse model of aging and dementia (Takahashi, 2010; Pacesova et al., 2022). Meanwhile, other AD animal models such as APP/PS1 transgenic mouse, 3XTg AD mouse, and 5XFAD mouse are transgenic mice that mimic histological damages in the brain involved in Aβ or Tau, not reflecting aging-related changes. The previous studies demonstrated increased oxidative stress, neuroinflammation, cognitive decline, neuronal injury, synaptic injuries, and Aβ deposition in the brain of 6-month-old SAMP8 mice (Takahashi, 2010). Therefore, SAMP8 was selected as a good animal model to study the effects of CSS on aging-related mild cognitive impairment as well as progressive AD in the current study. The 5-month-old SAMP8 mice and SAMR1 mice were orally administered CSS or vehicle for 8 weeks in this study, respectively. Consistent with the previous report by Takahashi (2010), impaired memory ability was observed in vehicle-treated SAMP8 mice at the end of experiments, compared with age-matched SAMR1 mice. In addition, CSS pretreatment was able to significantly improve the learning function of aged SAMP8 mice in the Morris water maze examination. Meanwhile, CSS pretreatment ameliorated the neuropathological alterations, including neuronal injury, synaptic structure damage, and Aβ deposition in the brain of SAMP8 mice, compared with vehicle-treated SAMP8 control mice. These findings indicated that CSS pretreatment could ameliorate or prevent aging-related cognitive impairment in the aging brain.

Increasing pieces of evidence implied that the impairment of the microbiota–gut–brain axis was involved in the pathogenesis of aging-related cognitive deterioration (Kohler et al., 2016; Yang et al., 2020). Imbalanced microbiota has been identified in the gut of AD patients and animal models. Furthermore, bacteria have also been observed in the brain of AD patients, indicating that microorganisms may be an accountable contributor to the pathogenesis and neuro-injuries of AD. Accumulating reports revealed that microbiota imbalances were capable of triggering intestinal inflammatory reactions and leading to intestinal barrier destruction. These events accelerate the entrance of gut bacteria-derived pathogens and toxins such as lipopolysaccharides into the circulatory system, followed by blood–brain barrier damage and neuroinflammation (Cani et al., 2007; Jin et al., 2013). In order to decipher the mechanisms by which CSS improved cognitive dysfunction in aging, we examined the effects of CSS prevention on the gut microbiota profiles by 16S rRNA gene analysis. Consistent with previous studies, the changes in the gut microbiota were detected in vehicle-treated SAMP8 mice, compared with age-matched SAMR1 controls. Elevated relative abundance of *Staphylococcus* and decreased relative abundance of *Lactobacillus* were observed in the gut of aged SAMP8 mice. Furthermore, at the species level, the relative abundance of *S. xylosus* increased while *L. reuteri* decreased. Interestingly, CSS pretreatment significantly decreased the abundance of *S. xylosus* and restored the abundance of *L. reuteri*. These results suggested that CSS probably reduced neuron damage and neuroinflammation by influencing microbes in the gut of aged mice.

CSS has been widely used as an alternative treatment for gastrointestinal diseases such as chronic gastritis, dyspepsia, and reflux esophagitis (Yang et al., 2013; Zhou et al., 2020). Recent reports showed CSS may have therapeutic effects on several neurologic disorders, including depression and anxiety (Yu et al., 2020; Han et al., 2021; Ma et al., 2022). The mechanisms involved in the neuroprotective effects of CSS included targeting the hippocampal 5-HT receptor and brain-derived neurotrophic factor, etc. (Yang et al., 2016). In addition, emerging pieces of evidence elucidated that the neuroprotective actions of CSS were also associated with its modulatory effects on gut microbiota composition. Decreased abundance of *P. distasonis*, *Firmicutes*, and *Proteobacteria* and increased *Desulfovibrionaceae* family members were apparent in CSS-treated depression mice (Ma et al., 2022). Consistent with the previous reports, the present study observed that CSS was able to increase the relative abundance of probiotic *Lactobacillus*, especially *L. reuteri*, and decrease harmful bacteria *Staphylococcus*, especially *S. xylosus*. These data indicated that the neuroprotective effects of CSS were associated with its modulation of the microbiota–gut–brain axis deficits in aged SAMP8 mice.

There were still some limitations in the present study. First, limited numbers of SAMP8 mice and SAMR1 mice were engaged to investigate the 16S rRNA amplicon sequencing data. Second, finding the efficacy of CSS on cognitive deficits and the microbiota–gut–brain axis was the first attempt of the present study, during which the complex processes of AD pathology might have caused some unexpected problems. Third, the potential causal effects of how CSS acted on cognitive deficits and the microbiota–gut–brain axis, such as the involvement of anti-neuroinflammatory effects and metabolism regulatory effects, were necessary. In addition, the involvement of neuroinflammation and the microbiota–gut–brain axis in the neuroprotective effects of CSS should be further researched in the future.

In summary, the current study provided scientific evidence that CSS possibly modulated gut microbiota imbalance, leading to the preventive effects on cognitive dysfunction in aging. This study paved the way for the development of a gut microbiota-targeted strategy for the prevention and treatment of aging-related cognitive decline.

## Data Availability

The original contributions presented in the study are included in the article/Supplementary Material; further inquiries can be directed to the corresponding authors.

## References

[B1] Abraham D. Feher J. Scuderi G. L. Szabo D. Dobolyi A. Cservenak M. (2019). Exercise and probiotics attenuate the development of Alzheimer's disease in transgenic mice: Role of microbiome. Exp. Gerontol. 115, 122–131. 10.1016/j.exger.2018.12.005 30529024

[B2] Akbari E. Asemi Z. Daneshvar Kakhaki R. Bahmani F. Kouchaki E. Tamtaji O. R. (2016). Effect of probiotic supplementation on cognitive function and metabolic status in alzheimer's disease: A randomized, double-blind and controlled trial. Front. Aging Neurosci. 8, 256. 10.3389/fnagi.2016.00256 27891089 PMC5105117

[B3] Athari Nik Azm S. Djazayeri A. Safa M. Azami K. Ahmadvand B. Sabbaghziarani F. (2018). Lactobacilli and bifidobacteria ameliorate memory and learning deficits and oxidative stress in beta-amyloid (1-42) injected rats. Appl. Physiol. Nutr. Metab. 43 (7), 718–726. 10.1139/apnm-2017-0648 29462572

[B4] Cani P. D. Amar J. Iglesias M. A. Poggi M. Knauf C. Bastelica D. (2007). Metabolic endotoxemia initiates obesity and insulin resistance. Diabetes 56 (7), 1761–1772. 10.2337/db06-1491 17456850

[B5] Cattaneo A. Cattane N. Galluzzi S. Provasi S. Lopizzo N. Festari C. (2017). Association of brain amyloidosis with pro-inflammatory gut bacterial taxa and peripheral inflammation markers in cognitively impaired elderly. Neurobiol. Aging 49, 60–68. 10.1016/j.neurobiolaging.2016.08.019 27776263

[B6] Cheng Y. Tian D. Y. Chen S. H. Ren J. R. Sun H. L. (2023). Physiological beta-amyloid clearance by the liver and its therapeutic potential for Alzheimer's disease. Acta Neuropathol. 23, 2559. 10.1007/s00401-023-02559-z 36964213

[B7] Cong L. Ren Y. Wang Y. Hou T. Dong Y. Han X. (2023). Mild cognitive impairment among rural-dwelling older adults in China: A community-based study. Alzheimers Dement. 19 (1), 56–66. 10.1002/alz.12629 35262288 PMC10078715

[B8] del Valle J. Bayod S. Camins A. Beas-Zárate C. Velázquez-Zamora D. A. González-Burgos I. (2012). Dendritic spine abnormalities in hippocampal CA1 pyramidal neurons underlying memory deficits in the SAMP8 mouse model of Alzheimer's disease. J. Alzheimers Dis. 32 (1), 233–240. 10.3233/JAD-2012-120718 22776969

[B9] Edgar R. C. (2013). Uparse: Highly accurate OTU sequences from microbial amplicon reads. Nat. Methods 10 (10), 996–998. 10.1038/nmeth.2604 23955772

[B10] GBD 2019 Dementia Forecasting Collaborators (2022). Estimation of the global prevalence of dementia in 2019 and forecasted prevalence in 2050: An analysis for the global burden of disease study 2019. Lancet Public Health 7 (2), e105–e125. 10.1016/S2468-2667(21)00249-8 34998485 PMC8810394

[B36] Guo T. Zhang D. Zeng Y. Huang T. Y. Xu H. Zhao Y.(2020). Molecular and cellular mechanisms underlying the pathogenesis of Alzheimer's disease. Mol Neurodegener 15, 40. 10.1186/s13024-020-00391-7 32677986 PMC7364557

[B11] Han S. K. Kim J. K. Park H. S. Shin Y. J. Kim D. H. (2021). Chaihu-Shugan-San (Shihosogansan) alleviates restraint stress-generated anxiety and depression in mice by regulating NF-κB-mediated BDNF expression through the modulation of gut microbiota. Chin. Med. 16 (1), 77. 10.1186/s13020-021-00492-5 34391441 PMC8364688

[B12] Huang Z. Lin H. W. K. Zhang Q. Zong X. (2022). Targeting alzheimer's disease: The critical crosstalk between the liver and brain. Nutrients 14 (20), 4298. 10.3390/nu14204298 36296980 PMC9609624

[B13] Jia H. M. Yu M. Zhang H. W. Zou Z. M. (2017). Chaihu-Shu-Gan-San regulates phospholipids and bile acid metabolism against hepatic injury induced by chronic unpredictable stress in rat. J. Chromatogr. B Anal. Technol. Biomed. Life Sci. 1064, 14–21. 10.1016/j.jchromb.2017.08.003 28886478

[B14] Jin L. Nation R. L. Li J. Nicolazzo J. A. (2013). Species-dependent blood-brain barrier disruption of lipopolysaccharide: Amelioration by colistin *in vitro* and *in vivo* . Antimicrob. Agents Chemother. 57 (9), 4336–4342. 10.1128/AAC.00765-13 23796941 PMC3754325

[B15] Kohler C. A. Maes M. Slyepchenko A. Berk M. Solmi M. Lanctôt K. L. (2016). The gut-brain Axis, including the microbiome, leaky gut and bacterial translocation: Mechanisms and pathophysiological role in alzheimer's disease. Curr. Pharm. Des. 22 (40), 6152–6166. 10.2174/1381612822666160907093807 27604604

[B16] Kong Y. Peng Q. Lv N. Yuan J. Deng Z. Liang X. (2020). Paeoniflorin exerts neuroprotective effects in a transgenic mouse model of Alzheimer's disease via activation of adenosine A1 receptor. Neurosci. Lett. 730, 135016. 10.1016/j.neulet.2020.135016 32371159

[B17] Lee T. H. Park S. You M. H. Lim J. H. Min S. H. Kim B. M. (2016). A potential therapeutic effect of saikosaponin C as a novel dual-target anti-Alzheimer agent. J. Neurochem. 136 (6), 1232–1245. 10.1111/jnc.13515 26710244

[B18] Ma C. Yuan D. Renaud S. J. Zhou T. Yang F. Liou Y. (2022). Chaihu-shugan-san alleviates depression-like behavior in mice exposed to chronic unpredictable stress by altering the gut microbiota and levels of the bile acids hyocholic acid and 7-ketoDCA. Front. Pharmacol. 13, 1040591. 10.3389/fphar.2022.1040591 36339629 PMC9627339

[B19] Pacesova A. Holubová M. Hrubá L. Strnadová V. Neprašová B. Pelantová H. (2022). Age-related metabolic and neurodegenerative changes in SAMP8 mice. Aging (Albany NY) 14 (18), 7300–7327. 10.18632/aging.204284 36126192 PMC9550245

[B20] Qin F. Liu J. Y. Yuan J. H. (2013). Chaihu-Shugan-San, an oriental herbal preparation, for the treatment of chronic gastritis: A meta-analysis of randomized controlled trials. J. Ethnopharmacol. 146 (2), 433–439. 10.1016/j.jep.2013.01.029 23376045

[B21] Rieder R. Wisniewski P. J. Alderman B. L. Campbell S. C. (2017). Microbes and mental health: A review. Brain Behav. Immun. 66, 9–17. 10.1016/j.bbi.2017.01.016 28131791

[B22] Takahashi R. (2010). Anti-aging studies on the senescence accelerated mouse (SAM) strains. Yakugaku Zasshi 130 (1), 11–18. 10.1248/yakushi.130.11 20046059

[B23] Tamtaji O. R. Heidari-Soureshjani R. Mirhosseini N. Kouchaki E. Bahmani F. Aghadavod E. (2019). Probiotic and selenium co-supplementation, and the effects on clinical, metabolic and genetic status in alzheimer's disease: A randomized, double-blind, controlled trial. Clin. Nutr. 38 (6), 2569–2575. 10.1016/j.clnu.2018.11.034 30642737

[B24] Vogt N. M. Kerby R. L. Dill-McFarland K. A. Harding S. J. Merluzzi A. P. Johnson S. C. (2017). Gut microbiome alterations in Alzheimer's disease. Sci. Rep. 7 (1), 13537. 10.1038/s41598-017-13601-y 29051531 PMC5648830

[B25] Wang M. Ren Q. Shi Y. Shu H. Liu D. Gu L. (2022). The effect of Alzheimer's disease risk factors on brain aging in normal Chineses: Cognitive aging and cognitive reserve. Neurosci. Lett. 771, 136398. 10.1016/j.neulet.2021.136398 34923042

[B26] Wang Y. Fan R. Huang X. (2012). Meta-analysis of the clinical effectiveness of traditional Chinese medicine formula Chaihu-Shugan-San in depression. J. Ethnopharmacol. 141 (2), 571–577. 10.1016/j.jep.2011.08.079 21933701

[B27] Yang N. Jiang X. Qiu X. Hu Z. Wang L. Song M. (2013). Modified chaihu shugan powder for functional dyspepsia: meta-analysis for randomized controlled trial. Evid. Based Complement. Altern. Med. 2013, 791724. 10.1155/2013/791724 PMC3666434 23762161

[B28] Yang P. Li L. Liu X. J. Cai X. Sun M. Z. He J. F. (2016). Effect of Chaihu-Shugan-San on the mRNA expression of the 5-HT1A receptor and cellular proliferation in the hippocampus of epileptic rats with depression. Exp. Ther. Med. 11 (1), 124–130. 10.3892/etm.2015.2867 26889228 PMC4726879

[B29] Yang X. Yu D. Xue L. Li H. Du J. (2020). Probiotics modulate the microbiota-gut-brain axis and improve memory deficits in aged SAMP8 mice. Acta Pharm. Sin. B 10 (3), 475–487. 10.1016/j.apsb.2019.07.001 32140393 PMC7049608

[B30] Yu M. Jia H. M. Zhang T. Shang H. Zhang H. W. Ma L. Y. (2020). Gut microbiota is the key to the antidepressant effect of Chaihu-Shu-Gan-San. Metabolites 10 (2), 63. 10.3390/metabo10020063 32050718 PMC7074034

[B31] Zeng Q. Li L. Siu W. Jin Y. Cao M. Li W. (2019). A combined molecular biology and network pharmacology approach to investigate the multi-target mechanisms of Chaihu Shugan San on Alzheimer's disease. Biomed. Pharmacother. 120, 109370. 10.1016/j.biopha.2019.109370 31563815

[B32] Zhang H. R. Peng J. H. Cheng X. B. Shi B. Z. Zhang M. Y. Xu R. X. (2015). Paeoniflorin atttenuates amyloidogenesis and the inflammatory responses in a transgenic mouse model of alzheimer's disease. Neurochem. Res. 40 (8), 1583–1592. 10.1007/s11064-015-1632-z 26068144

[B33] Zhao W. (2012). Effect of Chaihu Shugan San on memory function in Alzheimer's disease rats (In Chinese). Chin. J. Exp. Traditional Med. Formulae 18 (10), 207–210.

[B34] Zhou Y. Zeng Z. Dong X. Fei J. Li B. (2020). Effects of chaihu-shugan-san for reflux esophagitis: A protocol for systematic review and meta-analysis. Med. Baltim. 99 (49), e23458. 10.1097/MD.0000000000023458 PMC7717765 33285744

[B35] Zhuang Z. Q. Shen L. L. Li W. W. Fu X. Zeng F. Gui L. (2018). Gut microbiota is altered in patients with alzheimer's disease. J. Alzheimers Dis. 63 (4), 1337–1346. 10.3233/JAD-180176 29758946

